# Regorafenib and metronomic capecitabine, cyclophosphamide, and aspirin in refractory metastatic colorectal cancer: results from the REPROGRAM-01 single-arm phase II trial

**DOI:** 10.1016/j.esmogo.2025.100270

**Published:** 2025-12-03

**Authors:** A. El Kaddissi, A. Vienot, J.-D. Fumet, A. Meurisse, T. Nguyen, E. Klajer, A. Bouard, L. Spehner, F. Fein, M. Stouvenot, E. Dochy, M. Rebucci-Peixoto, H. Almotlak, J. Henriquez, Z. Selmani, D. Vernerey, E. Hervouet, A. Folletet, S. Kim, F. Ghiringhelli, C. Borg

**Affiliations:** 1 Department of Medical Oncology, University Hospital of Besançon, Besançon, France; 2 University of Franche-Comté, EFS, INSERM, UMR RIGHT, Besançon, France; 3 Clinical Investigational Center, CIC-1431, Besançon, France; 4 Department of Medical Oncology, Centre Georges-François Leclerc, Dijon, France; 5 ITAC Platform, University of Franche-Comté, Besançon, France; 6 Department of Gastroenterology, University Hospital of Besançon, Besançon, France; 7 Bayer SA-NV, Brussels, Belgium; 8 Department of Oncobiology, University Hospital of Besançon, Besançon, France

**Keywords:** colorectal cancer, regorafenib, metronomic chemotherapy, angiopoietin-2, NPY ctDNA

## Abstract

**Background:**

Treatment of chemotherapy-resistant metastatic colorectal cancers (mCRCs) is still an unmet medical need. Combining regorafenib, an antiangiogenic multikinase inhibitor with metronomic chemotherapy and aspirin may enhance efficacy to circumvent the tumor microenvironment and offer better clinical outcomes. REPROGRAM-01 was a signal-seeking phase II study assessing the efficacy and safety of multimodal metronomic chemotherapy combined with regorafenib (NCT04534218).

**Patients and methods:**

mCRC patients involved were previously exposed to conventional chemotherapies, bevacizumab, anti-epidermal growth factor receptor (anti-EGFR) if *RAS* wild type, and pembrolizumab if deficient mismatch repair disease. Regorafenib (starting dose 80 mg/day orally with weekly escalation, per 40 mg increment, to 160 mg/day) was combined with multimodal metronomic chemotherapy including capecitabine (625 mg/m^2^ twice daily continuously) and cyclophosphamide (50 mg daily) for 6 months plus aspirin (75 mg once daily). The primary endpoint was the objective response rate (ORR). Secondary endpoints were overall survival (OS), progression-free survival (PFS), safety, and evaluation of exploratory biomarkers.

**Results:**

Forty-eight patients were included. No unexpected serious adverse event was reported and 10 patients discontinued regorafenib for toxicity. The most common grade 3 and 4 toxicities involved palmoplantar erythrodysesthesia (14.6%), diarrhea (6.3%), hypertension (4.2%), and lymphopenia (4.2%). The best ORR was 4.2% [95% confidence interval (CI) 0.8% to 12.5%]. The trial was negative on the primary endpoint aiming to achieve an ORR of 15%, compared with 4% for null hypothesis. Median PFS and OS were 4.7 months (95% CI 3.7-5.9 months) and 9.5 months (95% CI 7.2-14.9 months), respectively. The PFS rates at 6 months and 12 months were 32.5% and 6.5%, respectively. A decrease of NPY methylated circulating tumor DNA >50% occurred in 16 out of the 26 assessable patients, leading to a median PFS of 5.5 months (95% CI 3.7-9.5 months) versus 3.6 (95% CI 1.8-9.6 months). A median PFS of 7.2 months (95% CI 3.7-11.6 months) was achieved in patients with decreasing angiopoietin-2 (ANG2) levels compared with 3.9 months (95% CI 1.3-19.4 months) in ANG2 nonresponding patients.

**Conclusions:**

According to the REPROGRAM-01 study, combining multimodal metronomic chemotherapy with regorafenib would double the number of patients who could benefit from regorafenib treatment without increasing toxicities.

## Highlights

• Treatment of chemotherapy-refractory colorectal cancers remains an unmet medical need.
• Metronomic administration of chemotherapy is an attractive approach to developing novel combinations with regorafenib.
• Metronomic chemotherapy plus regorafenib doubled the PFS achieved with antiangiogenic tyrosine kinase inhibitors.
• Mmetronomic chemotherapy plus regorafenib increased survival in patients displaying decreased plasmatic ctDNA and ANG2 levels.

## Introduction

Despite advances in treatment, metastatic colorectal cancer (mCRC) continues to present a significant therapeutic challenge, with limited options available for patients who have exhausted standard treatments such as 5-fluorouracil, irinotecan, oxaliplatin, anti-vascular endothelial growth factor (anti-VEGF), and anti-epidermal growth factor receptor (anti-EGFR) therapies.

Regorafenib, an orally administered multikinase inhibitor, has emerged as a promising agent for treatment-resistant mCRC. Its mechanism of action targets multiple pathways associated with tumor angiogenesis (VEGFR1-3, TIE2), oncogenesis (KIT, RET), and the tumor microenvironment [platelet-derived growth factor receptor (PDGFR)], which are implicated in the progression of colorectal tumors. The efficacy of regorafenib was first demonstrated in the CORRECT trial, a phase III, multicenter, placebo-controlled study involving 760 patients, which showed a significant improvement in overall survival (OS) for regorafenib-treated patients compared with placebo (6.4 months versus 5.0 months). Median progression-free survival (PFS) was 1.9 months [interquartile range (IQR) 1.6-3.9 months] in the regorafenib group and 1.7 months (IQR 1.4-1.9 months) in the placebo group. This trial established regorafenib as the first targeted therapy to provide a survival benefit in chemotherapy-refractory mCRC, despite the occurrence of grade 3-4 adverse events such as hand–foot skin reactions and fatigue.^1^

To address the generalizability of these findings, the CONCUR trial was carried out. Conducted across multiple Asian countries, this trial similarly demonstrated that regorafenib significantly prolonged OS (8.8 months in the regorafenib group versus 6.3 months in the placebo group) and improved PFS (3.2 months versus 1.7 months).^2^

The ReDOS study aimed to determine whether starting at a lower regorafenib dose and gradually increasing it could reduce side-effects while maintaining therapeutic effectiveness.^3^ Forty-three percent of patients in the dose-escalation group completed two cycles and continued to a third cycle, compared with 26% in the standard-dose group. Median PFS and OS were 2.8 months and 9.8 months in the dose-escalation group.

To improve regorafenib efficacy, combinations with chemotherapies were investigated. Regorafenib added toxicities to FOLFOX or 5-fluorouracil and oxaliplatin or irinotecan in mCRC patients, but generated no significant effect on 5-fluorouracil pharmacokinetics.^4^ The addition of regorafenib to 5-fluorouracil and oxaliplatin or irinotecan as second-line therapy for mCRC led to an increase in objective response rates (ORR) and PFS but the severe toxicities reported limited the magnitude of the clinical benefit.^5^

In addition, treatment of CRC that have become refractory to conventional chemotherapy remains an important issue for patients. The organization of a complex microenvironment could partly account for the acquisition of such therapeutic resistance.^6^ Metronomic chemotherapy, characterized by the frequent, low-dose administration of cytotoxic drugs, is designed to minimize adverse effects while maintaining consistent inhibition on tumor and stromal cell interactions.^7^ This approach has shown promise in preclinical and clinical studies, suggesting potential benefits in terms of antiangiogenic effects, immunomodulation, and tumor growth inhibition.^8^^,^^9^ Low dose and continuous administration of chemotherapy was shown to circumvent angiogenesis.^10^ Daily administration of cyclophosphamide at low dose decreased regulatory T-cell (Treg) levels and functions.^11^^,^^12^ Continuous administration of 5-fluorouracil has also been shown to deplete myeloid-derived suppressor cells in rodent models and patients.^13^^,^^14^ Metronomic chemotherapy is an option for the treatment of metastatic breast cancer^15^, ^16^, ^17^, ^18^ and the validated adjuvant treatment of nasopharyngeal carcinoma.^19^ In CRC, metronomic administration of capecitabine in combination with bevacizumab is a validated treatment in the first-line metastatic setting.^20^

Aspirin might reduce thymidylate phosphorylase and limit platelet and macrophage-mediated angiogenesis.^21^ In parallel, the COX2 inhibition by aspirin has been shown to deplete prostaglandin E2 (PGE2) levels in a previous pharmacokinetic study.^22^ Aspirin was previously suggested as potentially effective in association with capecitabine in locally advanced rectal carcinoma and in heavily pretreated mCRC patients.^23^^,^^24^ However, a limited number of studies have investigated the potential interest to combine aspirin and antiangiogenic multikinase inhibitors.^25^

Therefore, the REPROGRAM-01 study was designed as a signal-seeking phase II study to assess the effectiveness and tolerance of regorafenib combined with multimodal metronomic chemotherapy, specifically low-dose capecitabine, cyclophosphamide, and aspirin.

## Methods

### Study design and participants

The REPROGRAM-01 study (NCT04534218) was a multicenter, open-label, proof-of-concept single-arm phase II trial to evaluate the efficacy and safety of the combination of multimodal metronomic chemotherapy and regorafenib in patients with chemorefractory mCRC. The primary objective was to assess the efficacy of this combination in terms of best ORR during the treatment period assessed by RECIST 1.1 criteria. Secondary objectives were to evaluate safety, PFS, OS, and health-related quality of life (HRQoL). The exploratory translational objective was to characterize the association of biomarkers of interest (circulating tumor DNA [ctDNA], angiogenic-related biomarkers) with outcomes.

Eligible patients were included in three centers across France and had histologically proven mCRC in progression after previous standard treatments (5-fluorouracil, irinotecan, oxaliplatin, anti-VEGF, anti-EGFR if *KRAS/NRAS* wild type, encorafenib plus anti-EGFR if *BRAF* V600E mutation, and immunotherapy if deficient mismatch repair [dMMR)]), or were not considered as candidates for these treatments. A life expectancy of ≥3 months was required. Patients aged ≥18 years were also eligible if they had an Eastern Cooperative Oncology Group Performance Status (ECOG-PS) of 0 or 1, adequate biological function (an absolute neutrophil count ≥1500 cells per mm^3^; platelet count ≥100 000 cells per mm^3^; creatinine clearance [according to Cockcroft formula] ≥50 ml/min; aspartate aminotransferase and alanine aminotransferase ≤2.5 × upper limit of normal [ULN; or ≤5 × ULN in the case of known liver metastases]; total bilirubin ≤2.5 × ULN, and proteinuria <2+ [dipstick urinalysis] or ≤1 g/24 h), and measurable disease according to RECIST 1.1. Main exclusion criteria included prior exposure to regorafenib, prior treatment with an investigational drug within 28 days before the start of study treatment, major surgical procedure within 28 days before initiation of study treatment, inadequate cardiac or respiratory function, known active central nervous system metastases and/or carcinomatous meningitis, and known dihydropyrimidine dehydrogenase deficiency.

The study was developed and promoted by the University Hospital of Besançon. The trial protocol was approved by the Ile de France III French Committee for Protection of Persons on 16 June 2020, and by the French Health Products Safety Agency on 19 August 2020. The study was conducted in accordance with the Declaration of Helsinki and the International Conference on Harmonization Good Clinical Practice guidelines and all patients provided written informed consent.

### Procedures

Regorafenib (STIVARGA®) was administered *per os* 3 weeks out of 4 weeks (one cycle corresponding to 4 weeks) until disease progression or unacceptable toxicity. For the first cycle, regorafenib was administered according to the ‘ReDOS’ schedule starting with 80 mg daily for the first week, followed by 120 mg and 160 mg daily for weeks 2 and 3, respectively. For subsequent cycles, regorafenib was administered at a dose of 80 mg, 120 mg, or 160 mg daily according to the toxicity observed with the last dose used in the first cycle. Metronomic chemotherapy was administrated *per os* as follows: cyclophosphamide 50 mg daily for 6 months; capecitabine 625 mg/m^2^ twice daily for 6 months; and aspirin 75 mg daily until disease progression.

Adverse events were measured according to the National Cancer Institute Common Terminology Criteria for Adverse Events (NCI CTCAE v5). Responses to treatment were assessed by RECIST 1.1 criteria. In the presence of liver metastases, Chun’s criteria have also been used to validate the response to antiangiogenic treatments: the objective response may be none, incomplete, or optimal.^26^ A scheduled blinded central review was carried out to assess radiologic response. Computed tomography scans were planned at baseline and every 8 weeks until disease progression. Follow-up visits were carried out every 3 months from the end-of-treatment visit until the end-of-study visit or death or loss to follow-up, whichever occurred first. Laboratory and adverse event monitoring were conducted at each visit.

### Statistical analysis

Regarding the primary objective, the primary endpoint was the best ORR defined by RECIST 1.1 criteria as the best disease response observed during the treatment period. PFS was defined as the time from the date of treatment initiation to disease progression in accordance with RECIST 1.1 or death from any cause (during the study treatment period or before the initiation of a new treatment), whichever occurred first. Alive patients without progression were censored at the last tumor assessment showing no progression during the study treatment period or before the initiation of a new treatment. OS was defined as the time from the treatment start date to death from any cause (patients who were alive were censored at the last follow-up visit). HRQoL was evaluated with European Organisation for Research and Treatment of Cancer (EORTC) QLC30 + CR29 and EQ-5D-3L questionnaires at baseline and every 2 months until disease progression. Five targeted dimensions were considered: global health, pain, physical functioning, fatigue, and emotional functioning. Biomarkers for exploratory translational objectives were the levels, at baseline and after 8 weeks, of plasmatic angiogenic-related biomarkers measured by enzyme-linked immunosorbent assay (ELISA) and ctDNA assessed by PCR, based on the determination of *WIF1* and *NPY* methylation in plasmatic cell-free DNA and considered as detectable if more than one DNA copy per ml was measured.^27^

The sample size was determined according to an A’Hern one-stage design with one-sided α and β risks of 0.05 and 0.2. Based on a theoretical 4% best ORR expected in the mCRC population when regorafenib is used in monotherapy^1^ and an expected 15% best ORR in our experimental phase II, 44 patients had to be included in the REPROGRAM-01 study. In this setting, the one-sided α and β risks were 0.05 and 0.2, respectively.

A Consolidated Standards of Reporting Trials (CONSORT) flow diagram of the progress describing enrollment, follow-up, end-of-treatment reason, and populations for analyses was proposed (Supplementary Figure S1, available at https://doi.org/10.1016/j.esmogo.2025.100270). Categorical variables were presented by absolute and relative frequencies and missing modality. Continuous variables were summarized using descriptive statistics, i.e. number of patients with available data (N); mean, median, and standard deviation (SD); 25%-75% quartile (Q1-Q3); and minimum and maximum. Follow-up was estimated using the reverse Kaplan–Meier method and was described using the median with its 95% confidence interval (95% CI). The Kaplan–Meier method was used to estimate time to event endpoints, the median, and event-free rates over time with 95% CI. Longitudinal analyses of HRQoL score changes compared with baseline were carried out with the mixed-model for repeated-measures, including time effects as a categorical variable. They were adjusted in relation to the baseline score, with the consideration of an unstructured covariance matrix. The model also included random effects on the intercept to reflect any individual deviance from the mean intercept. The relationship between the baseline value of ctDNA (in its continuous form) and PFS was modeled using the restricted cubic splines method to investigate other relevant cut-offs of interest beyond the positive/negative consideration. All statistical analyses were conducted using SAS® Version 9.4 (SAS Institute, Cary, NC) and R software version 4.3.0 (R Development Core Team, Vienna, Austria; http://www.r-project.org). The database lock for the present analysis was carried out on 3 December 2024.

### Role of the funding source

The funder had no role in the study design, data collection, analysis, interpretation, or preparation of the manuscript and decision to publish. All authors had final responsibility for the decision to submit for publication.

## Results

### Patients’ characteristics

Between November 2020 and December 2022, 48 patients were included at two university hospitals and one general hospital. Table 1 shows the baseline demographics, clinical characteristics, and follow-up of the patients. The median age was 64.1 years (minimum-maximum 32.0-83.1 years), nearly half of the patients (45.8%) were female, and 20 of them (41.7%) had metachronous metastatic disease. A total of 39 patients (81.3%) had liver metastases, 20 (41.7%) had lung metastases, and 4 (8.3%) had peritoneal metastases. Mutations in *RAS* oncogenes were reported in 25 (52.1%) patients, 6 (12.8%) patients had a tumor with a *BRAF* V600E mutation, and 1 patient had a dMMR status. The median number of therapeutic lines previously received was 3 (minimum-maximum 1-7). At baseline, 29 (60%) patients had an ECOG-PS of 0 and 19 (40.4%) patients had an ECOG-PS of 1.

**Table 1:** Baseline patient demographics and clinical characteristics.

Characteristic	*n* (%)
Age, median years (minimum-maximum)	64.1 (32-83.1)
Male	26 (54.2)
Female	22 (45.8)
Performance status	
0	29 (59.6)
1	19 (40.4)
BMI, median (minimum-maximum)	24.6 (19.3-45.2)
Localization	
Right colon	9 (18.8)
Left colon	17 (35.4)
Colon transverse	4 (8)
Rectum	18 (37.5)
Metastases	
Synchronous	28 (58.3)
Metachronous	20 (41.7)
Metastatic sites, median (minimum-maximum)	1 (1-4)
Metastatic sites	
Lung	20 (41.7)
Liver	39 (81.3)
Bone	1 (2.1)
Lymph nodes	11 (22.9)
Peritoneum	4 (8.3)
Others (skin, spleen, ovaries)	3 (6.5)
*KRAS*	
Wild type	29 (60.4)
Mutated	19 (39.6)
*NRAS*	
Missing	1
Wild type	39 (86.7)
Mutated	6 (13.3)
*BRAF*	
Missing	1
Wild type	41 (87.2)
Mutated	6 (12.8)
Mismatch repair	
Missing	2
pMMR	45 (97.8)
dMMR	1 (2.2)
Prior metastasis surgery	
No	28 (58.3)
Yes	20 (41.7)
Prior chemotherapy for metastatic disease	48 (100)
Prior exposure to a biotherapy	45 (93.8)
Previous line of therapy	
1-2	17 (35.4)
3	13 (27.1)
≥4	18 (37.5)
Delay between the diagnosis of metastasis and inclusion, months, median (minimum-maximum)	24.6 (19.3-45.2)

dMMR, deficient mismatch repair; pMMR, proficient mismatch repair.

Two patients did not carry out the first assessment. The first had a disease-related occlusion before treatment initiation. A second patient died of bacterial lung disease not related to the experimental treatment during the third week of the first cycle. These two patients were included in the efficacy analysis and not in the safety-related analyses or the search for biomarkers of interest. The median follow-up for all patients was 20.0 months (95% CI 16.3-31.2 months).

### Safety and exposure

In the safety analysis population, 32 patients (66.7%) reported at least one grade 3 or 4 toxicity, related to the experimental therapy for 21 patients. Indeed, the most common grade 3 and 4 toxicities included palmoplantar erythrodysesthesia (*n* = 7, 14.6%), diarrhea (*n* = 3, 6.3%), lymphopenia (*n* = 2, 4.2%), and hypertension (*n* = 2, 4.16%). Grade 1/2 and 3 asthenia was exhibited by 43.8% and 2.1% of the patients, respectively. No additional bleeding or digestive toxicities were reported with the combination of aspirin. The toxicities observed are listed in Table 2.

**Table 2:** Summary of the treatment-related adverse events.

	Grade 1 and 2	Grade 3 and 4
	Number of patients, *n* (%)	
Palmoplantar erythrodysesthesia	37 (77.1)	7 (14.6)
Diarrhea	24 (50)	3 (6.3)
Hypertension	2 (4.1)	2 (4.2)
Lymphopenia	9 (18.75)	2 (4.2)
Anemia	5 (10.4)	1 (2.1)
Thrombopenia	6 (12.5)	—
Aspartate aminotransferase increased	4 (10.4)	1 (2.1)
Asthenia	21 (43.75)	1 (2.1)
Generalized exfoliative dermatitis	—	1 (2.1)
Increased bilirubin	3 (6.25)	1 (2.1)
Hypokalemia	7 (14.6)	1 (2.1)
Hyponatremia	—	1 (2.1)
Neutropenia	2 (4.1)	1 (2.1)
Proteinuria	2 (4.1)	1 (2.1)
Maculopapular skin reaction	1 (2.08)	1 (2.1)
Stomatitis	11 (22.9)	1 (2.1)
Dysphonia	8 (16.6)	—
Muscle contracture	7 (14.6)	—
Cutaneous dryness	7 (14.6)	—

**Figure 1:**
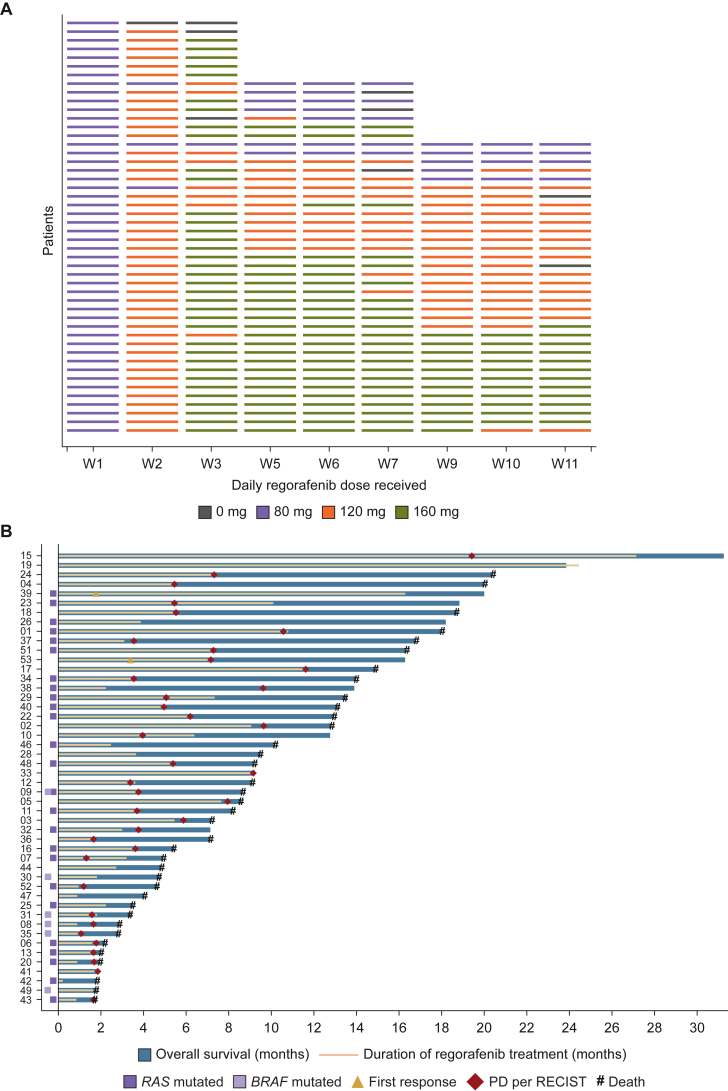
**Swimmer plot presenting dosing history (A) and clinical outcomes (B).** (A) Dosing history is presented up to week 3 of cycle 3. Individual patients are represented throughout the different weeks and cycles by a horizontal series of bars. (B) Swimmer plot of patient status according to the presence of *RAS/RAF* mutations and occurrence of objective responses. Each lane is color coded according to the duration of the treatment and the study follow-up until defined events. PD, progression disease; W, week.

In the intention-to-treat (ITT) analysis population, the median duration of regorafenib therapy was 3.5 months (minimum-maximum 0.2-27.1 months). Treatment was discontinued in 47 patients, due to progression in 28 patients (58.3%) and due to toxicity in 10 patients (20.8%). Figure 1A reports the weekly exposure to regorafenib during the first three cycles. Thirty-four patients (70.8%) who completed the first two cycles initiated cycle 3. At cycle 2 initiation, 23 (47.9%) patients received 160 mg of regorafenib and 12 (25%) patients received 120 mg. At cycle 3 initiation, 12 (25%) patients started at a dose of 160 mg while 17 (35.4%) patients received 120 mg daily. Interestingly, the addition of metronomic chemotherapy to regorafenib did not hamper the administration of the antiangiogenic tyrosine kinase inhibitor, as 38.8% and 18.5% of patients in the previously reported ReDOS trial started cycles 2 and 3, respectively, at a dose of 160 mg for regorafenib.^3^

### Efficacy

The individual profile of REPROGRAM-01 treatment for each patient is reported in Figure 1B. In the ITT population, the best ORR evaluated with RECIST 1.1 criteria was 4.2% (95% CI 0.8% to 12.5%, *n* = 2). As the lower limit of the 95% CI falls below the null hypothesis (4%), the trial did not meet its primary endpoint. Chun’s morphologic criteria were used for tumor evaluation in patients with liver metastases. Among the 33 patients with assessable liver metastases at inclusion, 18 (54.5%), 3 (9.1%), and 12 (36.4%) patients had absence, incomplete, and optimal responses, respectively. However, responses defined by Chun’s criteria were not correlated with PFS or OS.

**Figure 2:**
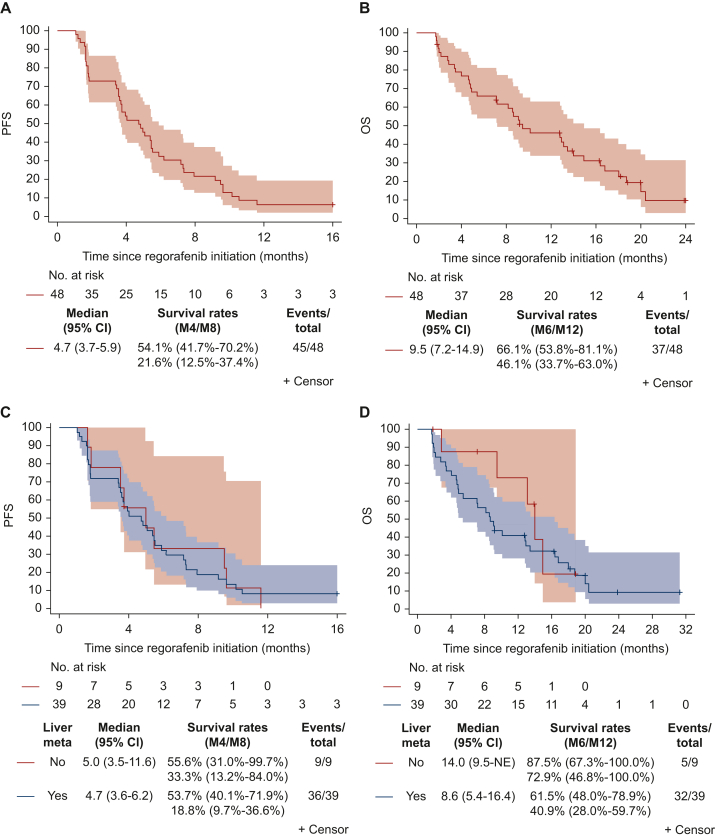
**Kaplan–Meier curves for progression-free survival (PFS) (A) and overall survival (OS) (B).** PFS (C) and OS (D) are also depicted according to the presence of liver metastases. CI, confidence interval; meta, metastases; NE, not estimable.

Median PFS was 4.7 months (95% CI 3.7-5.9 months). The PFS rates at 4 months and 8 months were 54.1% (95% CI 41.7% to 70.2%) and 21.6% (95% CI 12.5% to 37.4%), respectively (Figure 2A). Median OS was 9.5 months (95% CI 7.2-14.9 months). The 6- and 12-month survival rates were 66.1% (95% CI 53.8% to 81.1%) and 46.1% (95% CI 33.7% to 63.0%), respectively (Figure 2B). Two out of the six patients from the subset of *BRAF*-mutated mCRC had a PFS >3 months. Conversely, the presence of *RAS* mutations appeared not to be associated with outcomes in patients treated with regorafenib and multimodal metronomic chemotherapy combination: median PFS of 3.7 months (95% CI 3.5-6.2 months) in *RAS-*mutated patients versus 5.5 months (95% CI 3.9-9.2 months) in *RAS* wild-type patients. Interestingly, our results suggest that regorafenib combined with multimodal metronomic chemotherapy might be an effective therapeutic option in patients with liver metastases (Figure 2C and D). The PFS achieved in the REPROGRAM-01 study was similar in patients with or without liver metastases [5.0 months (95% CI 3.5-11.6 months) versus 4.7 months (95% CI 3.6-6.2 months)].

Results for the time until the definitive deterioration of the quality of life are proposed in Supplementary Figures S2 and S3, available at https://doi.org/10.1016/j.esmogo.2025.100270. As expected, the patient’s HRQoL in the five targeted dimensions was deteriorated at the first evaluation after the beginning of the treatment without any substantial improvement on further evaluations.

### Translational analyses

#### ctDNA monitoring

**Figure 3:**
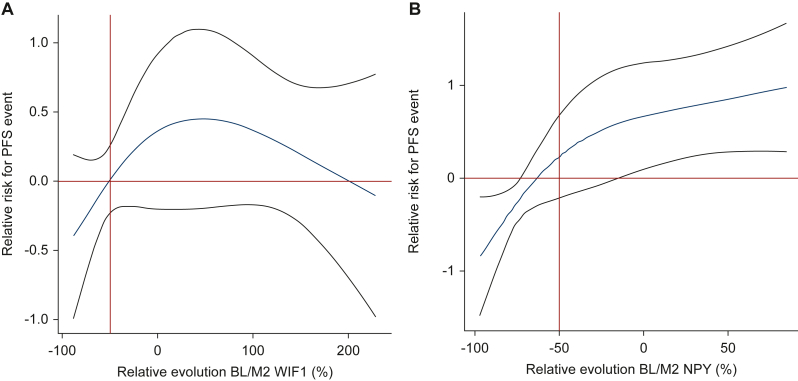

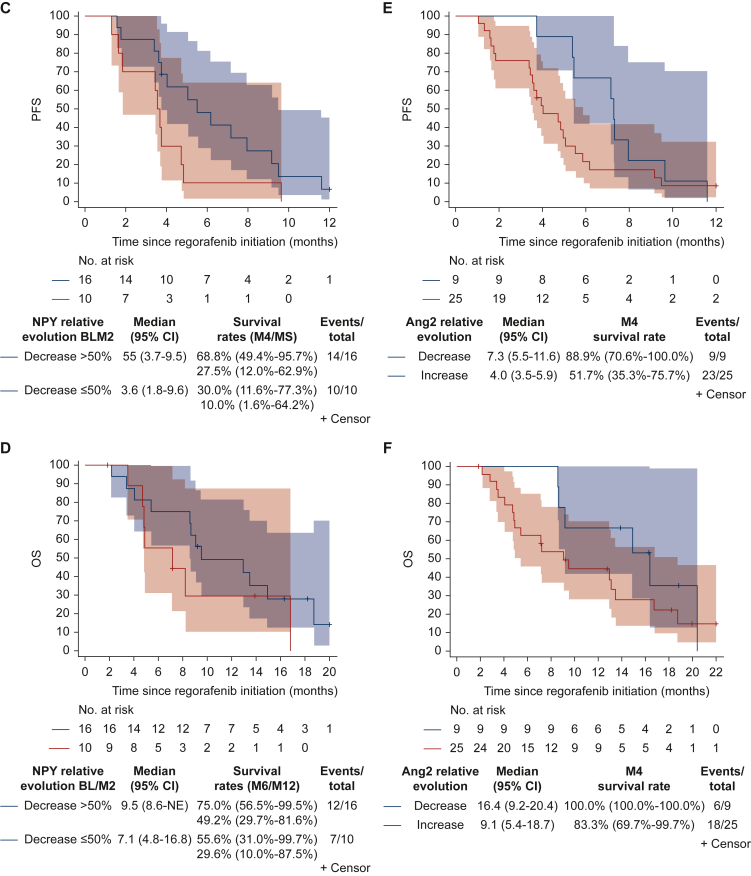
**Influence of ctDNA and angiogenic-related biomarkers on regorafenib and multimodal metronomic chemotherapy effectiveness.** Restricted cubic spline method was used to define the relation between the dynamic evolution of methylated ctDNA levels {[(methylated ctDNA level at M2 – methylated ctDNA level at BL)/methylated ctDNA level at BL] × 100} and the relative risk for PFS event for *WIF1* (A) and *NPY* (B). Kaplan–Meier curves describing PFS (C) and OS (D) for patients with a NPY methylated-ctDNA level decrease >50% after treatment. Kaplan–Meier estimation of the PFS (E) and OS (F) for patients displaying an increase or a decrease of angiopoietin-2 (ANG2) levels after treatment. BL, baseline; CI, confidence interval; ctDNA, circulating tumor DNA; M2, month 2; OS, overall survival; PFS, progression-free survival.

We measured methylated *WIF1* and *NPY* plasmatic DNA sequences in 42 patients at baseline. *WIF1* and *NPY* methylated ctDNA levels were monitored in 29 and 26 patients, respectively, after 2 months of therapy. The median methylated DNA at baseline was 4.5 copies per ng of bisulfited DNA (minimum-maximum 0-107.1 copies per ng) for *WIF1* and 2.9 copies/ng (minimum-maximum 0.5-92.2 copies/ng) for *NPY*. *WIF1* and *NPY* detection levels were correlated with PFS and OS at baseline (Supplementary Figure S4, available at https://doi.org/10.1016/j.esmogo.2025.100270). Regorafenib and metronomic chemotherapy decreased *WIF1* and *NPY*-ctDNA levels in 17 and 20 patients, respectively. *WIF1* and *NPY* methylated DNA level cut-offs were validated using the restricted cubic splines method with graphical evaluation. Interestingly, the decline of *NPY* but not *WIF1* methylated ctDNA after treatment better predicted PFS (Figure 3A and B). The subgroup of patients for whom treatment with multimodal metronomic chemotherapy and regorafenib induced a decrease in *NPY*-ctDNA of >50% achieved a median PFS of 5.5 months (95% CI 3.7-9.5 months) versus 3.6 months (95% CI 1.8-9.6 months) and a median OS of 9.5 months [95% CI 8.6 months-not estimable (NE)] versus 7.1 months (95% CI 4.8-16.8 months; Figure 3C and D).

#### Angiogenic-related biomarkers

We have previously reported a role for angiopoietin-2 (ANG2) and stanniocalcin-1 (STC1) in predicting clinical outcomes in mCRC.^28^^,^^29^ The angiogenic growth factors ANG2, STC1, and VEGF were then measured in 34 patients with available plasma samples at baseline and 2 months after treatment initiation. The combination of regorafenib and multimodal metronomic chemotherapy was able to decrease VEGF, STC1, and ANG2 levels in 22 (64.7%), 21 (61.8%), and 9 (26.4%) patients, respectively. The decline of VEGF or STC1 was not correlated with PFS and OS. Conversely, a decrease in ANG2 predicted the benefit of the combination. The probability of achieving PFS after 4 months of therapy was 87.5% for patients with a decrease in ANG2 compared with 48% for other patients. Median PFS was 7.2 months (95% CI 3.7-11.6 months) in patients with decreasing ANG2 levels compared with 3.9 months (95% CI 1.3-19.4 months; Figure 3E). Median OS was 16.4 months (95% CI 9.2-20.4 months) in patients with decreasing ANG2 levels compared with 9.1 months (95% CI 5.4-18.7 months; Figure 3F).

## Discussion

Despite the negative result for the primary objective of the study based on the best ORR, results reported in the REPROGRAM-01 study provide evidence that the addition of metronomic chemotherapy to regorafenib is feasible and appears to provide clinical benefit to patients for the other efficacy outcomes.

The toxicity profile observed in the REPROGRAM-01 study is in line with the results presented in the pivotal CORRECT study.^1^ In fact, the rate of discontinuation due to toxicity in our study was 21.7%, compared with the 19.6% of discontinuation due to adverse events reported in the ReDOS study using the regorafenib dose-escalation regimen.^3^ The most common grade 3/4 toxicities in the CORRECT and ReDOS studies were hand–foot syndrome (17% and 15%, respectively), fatigue (10% and 13%), and diarrhea (8% and 2%). The combination of multimodal metronomic chemotherapy with regorafenib does not seem to change the profile of these adverse events (14.6% grade 3/4 hand–foot syndrome, 2.1% anemia, 6.3% diarrhea). Occurrence of asthenia here was 45%, in line with the incidence reported in pivotal studies.^1^, ^2^, ^3^

The efficacy analysis demonstrates that the use of regorafenib in combination with metronomic chemotherapy does not increase the number of partial responses (two patients, 4%) according to the RECIST 1.1 criteria. Nevertheless, the treatment regimen allows long-term disease control that is favorable compared with the CORRECT, CONCUR, and ReDOS studies.^1^, ^2^, ^3^ Indeed, the median PFS was 4.7 months (95% CI 3.7-5.9 months) in patients included in the REPROGRAM-01 study. PFS rates at 4 months and 8 months were 54.1% and 21.6%, respectively, with three patients treated >12 months. Strikingly, median PFS was 1.9 months and 2.8 months in the CORRECT and ReDOS studies, respectively, with <10% of the patients remaining free of progression after 6 months of follow-up. Of note, the presence of *BRAF* mutations was the only clinical and tumor-related determinant of efficacy in the REPROGRAM-01 study. No patient with a *BRAF*-mutated tumor achieved PFS >4 months. *PI3KCA*-activating mutations were observed in three patients. It is unlikely that *PI3KCA* status influenced the results since no ORR was reported in these patients and survival outcomes were similar to those of the whole population. Altogether, these clinical outcomes suggest a potent efficacy of regorafenib in combination with multimodal metronomic chemotherapy. An accurate prognosis model was validated in the REBECCA trial.^30^ In this study, OS was independently influenced by poor performance status, short time from initial diagnosis of metastases to the start of regorafenib, low initial regorafenib dose, more than three metastatic sites, presence of liver metastases, and *KRAS* mutations. Patients from the REBECCA study in the low, intermediate, and high risk of death groups displayed a median survival of 9.2 months, 5.2 months, and 2.5 months, respectively, versus 13.7 months (95% CI 9.1 months-NE), 13.1 months (95% CI 7.2-20.4 months), and 4.7 months (95% CI 3.4 months-NE) in the REPROGRAM-01 trial.

To consolidate these results, we carried out a dynamic analysis of the ctDNA under the experimental treatment. In their study published in 2023, Lee et al. investigated the predictive value of ctDNA dynamics for assessing the efficacy of regorafenib in patients with mCRC.^31^ By analyzing ctDNA samples collected at baseline, after two treatment cycles, and at progression, the authors used variant allele frequency (VAF) changes to estimate tumor burden, focusing particularly on the sum of VAF across detected mutations as a comprehensive marker. Their findings showed that patients with a ≥50% reduction in ctDNA burden after two cycles exhibited a notably improved PFS and OS. Specifically, those with this reduction in ctDNA had a median PFS of 6.1 months versus 2.7 months for patients with <50% reduction, and a median OS of 11.3 months versus 5.9 months.^31^ Similar results were reported using droplet PCR to monitor *RAS* mutations in ctDNA.^32^ In the REPROGRAM-01 study, ctDNA was determined by monitoring of the methylated *WIF1* and *NPY* sequences in plasma. A decrease in ctDNA was achieved in more than half of patients after two cycles of regorafenib. Interestingly, molecular responses determined by the assessment of *NPY* methylation status were correlated with PFS. In the absence of *NPY* molecular response, the combination of regorafenib and metronomic chemotherapy had a limited clinical impact: 23% of patients were free of progression after 4 months of therapy with a median PFS of 3.5 months of versus 53% 4-month PFS rate and 5.0 months of median PFS when *NPY*-ctDNA decreased. The correlation observed here between molecular responses as measured by *NPY* methylated ctDNA status and PFS supports the bioactivity of the combination therapy evaluated in the REPROGRAM-01 study.

Another translational insight from the REPROGRAM-01 study was the monitoring of angiogenic-related biomarkers. Investigators of the CORRECT study have assessed 15 proteins of interest in the plasma of mCRC patients exposed to regorafenib. VEGF and ANG2 levels had no association with clinical outcomes.^33^ The addition of multimodal metronomic chemotherapy decreased VEGF and STC1 levels in 46% and 48% of the patients in our study, respectively. However, these biological effects were not correlated with survival. Conversely, a decrease of ANG2 was noticed in 26.4% of the patients treated with the combination of regorafenib and metronomic chemotherapy. Our results pointed out a clear association of ANG2 modulation on PFS.

While the presence of patients with long-term clinical benefit and the biological correlates sustain the efficacy of the experimental strategy assessed here, our study also has limitations. Firstly, the expected number of objective responses was not observed and only 4% of patients had a response according to the RECIST 1.1 criteria, implying that the REPROGRAM-01 study did not achieve its primary endpoint. The initial choice of objective responses as a primary endpoint in the context of antiangiogenic and advanced-line therapy was inappropriate. However, it might be postulated that metronomic chemotherapy acts on the tumor microenvironment rather than on tumor cell viability, leading to an enhancement of regorafenib cytostatic activity, as suggested by the prolonged PFS observed here. Another limitation is the lack of a control arm enabling the validation of our hypothesis. However, the clinical characteristics of the patients included in the REPROGRAM-01 study, as well as the *RAS/RAF* mutation distribution, are comparable to those reported in the CORRECT trial. Of note, all patients were refractory to chemotherapy. Another limitation is that the REPROGRAM-01 study design makes it impossible to assess which component of the intervention is responsible for the activity signal seen in the PFS results. Our objective was to provide proof of concept of a potential clinical and biological activity in order to promote the development of this novel strategy, aimed at improving the antiangiogenic efficacy level while maintaining a safety profile compatible with the treatment of advanced mCRC. Therefore, these results prompted us to launch a confirmatory phase III trial to compare regorafenib monotherapy with regorafenib plus multimodal metronomic chemotherapy in chemorefractory patients (NCT06425133).

Altogether, the REPROGRAM-01 study pointed out that the addition of multimodal metronomic chemotherapy to regorafenib has a clinical efficacy leading to ctDNA molecular responses and durable PFS. A decrease in ANG2 plasmatic levels is a potential biomarker that correlates with regorafenib and metronomic chemotherapy efficacy.

## References

[1] Grothey A., Van Cutsem E., Sobrero A. (2013). Regorafenib monotherapy for previously treated metastatic colorectal cancer (CORRECT): an international, multicentre, randomised, placebo-controlled, phase 3 trial. Lancet.

[2] Li J., Qin S., Xu R. (2015). Regorafenib plus best supportive care versus placebo plus best supportive care in Asian patients with previously treated metastatic colorectal cancer (CONCUR): a randomised, double-blind, placebo-controlled, phase 3 trial. Lancet Oncol.

[3] Bekaii-Saab T.S., Ou F.S., Ahn D.H. (2019). Regorafenib dose-optimisation in patients with refractory metastatic colorectal cancer (ReDOS): a randomised, multicentre, open-label, phase 2 study. Lancet Oncol.

[4] Schultheis B., Folprecht G., Kuhlmann J. (2013). Regorafenib in combination with FOLFOX or FOLFIRI as first- or second-line treatment of colorectal cancer: results of a multicenter, phase Ib study. Ann Oncol.

[5] Sanoff H.K., Goldberg R.M., Ivanova A. (2018). Multicenter, randomized, double-blind phase 2 trial of FOLFIRI with regorafenib or placebo as second-line therapy for metastatic colorectal cancer. Cancer.

[6] Straussman R., Morikawa T., Shee K. (2012). Tumour micro-environment elicits innate resistance to RAF inhibitors through HGF secretion. Nature.

[7] Bocci G., Kerbel R.S. (2016). Pharmacokinetics of metronomic chemotherapy: a neglected but crucial aspect. Nat Rev Clin Oncol.

[8] Filippi R., Lombardi P., Depetris I. (2018). Rationale for the use of metronomic chemotherapy in gastrointestinal cancer. Expert Opin Pharmacother.

[9] Spehner L, Bouard A, El Kaddissi A (2026). Clinical and biological determinants of multimodal metronomic chemotherapy efficacy in chemo-refractory gastrointestinal cancers. Int J Cancer.

[10] Hanahan D., Bergers G., Bergsland E. (2000). Less is more, regularly: metronomic dosing of cytotoxic drugs can target tumor angiogenesis in mice. J Clin Invest.

[11] Laheurte C., Thiery-Vuillemin A., Calcagno F. (2020). Metronomic cyclophosphamide induces regulatory T cells depletion and PSA-specific T cells reactivation in patients with biochemical recurrent prostate cancer. Int J Cancer.

[12] Ghiringhelli F., Menard C., Puig P.E. (2007). Metronomic cyclophosphamide regimen selectively depletes CD4+CD25+ regulatory T cells and restores T and NK effector functions in end stage cancer patients. Cancer Immunol Immunother.

[13] Peereboom D.M., Alban T.J., Grabowski M.M. (2019). Metronomic capecitabine as an immune modulator in glioblastoma patients reduces myeloid-derived suppressor cells. JCI Insight.

[14] Vincent J., Mignot G., Chalmin F. (2010). 5-Fluorouracil selectively kills tumor-associated myeloid-derived suppressor cells resulting in enhanced T cell-dependent antitumor immunity. Cancer Res.

[15] Wildiers H., Tryfonidis K., Dal Lago L. (2018). Pertuzumab and trastuzumab with or without metronomic chemotherapy for older patients with HER2-positive metastatic breast cancer (EORTC 75111-10114): an open-label, randomised, phase 2 trial from the Elderly Task Force/Breast Cancer Group. Lancet Oncol.

[16] Bottini A., Generali D., Brizzi M.P. (2006). Randomized phase II trial of letrozole and letrozole plus low-dose metronomic oral cyclophosphamide as primary systemic treatment in elderly breast cancer patients. J Clin Oncol.

[17] Cazzaniga M.E., Vallini I., Montagna E. (2021). Metronomic chemotherapy (mCHT) in metastatic triple-negative breast cancer (TNBC) patients: results of the VICTOR-6 study. Breast Cancer Res Treat.

[18] Ellis G.K., Barlow W.E., Gralow J.R. (2011). Phase III comparison of standard doxorubicin and cyclophosphamide versus weekly doxorubicin and daily oral cyclophosphamide plus granulocyte colony-stimulating factor as neoadjuvant therapy for inflammatory and locally advanced breast cancer: SWOG 0012. J Clin Oncol.

[19] Chen Y.-P., Liu X., Zhou Q. (2021). Metronomic capecitabine as adjuvant therapy in locoregionally advanced nasopharyngeal carcinoma: a multicentre, open-label, parallel-group, randomised, controlled, phase 3 trial. Lancet.

[20] Simkens L.H.J., van Tinteren H., May A. (2015). Maintenance treatment with capecitabine and bevacizumab in metastatic colorectal cancer (CAIRO3): a phase 3 randomised controlled trial of the Dutch Colorectal Cancer Group. Lancet.

[21] Zhu G.H., Schwartz E.L. (2003). Expression of the angiogenic factor thymidine phosphorylase in THP-1 monocytes: induction by autocrine tumor necrosis factor-alpha and inhibition by aspirin. Mol Pharmacol.

[22] Boutaud O., Sosa I.R., Amin T. (2016). Inhibition of the biosynthesis of prostaglandin E2 by low-dose aspirin: implications for adenocarcinoma metastasis. Cancer Prev Res (Phila).

[23] Farrugia M.K., Long M.D., Mattson D.M. (2021). Concurrent aspirin use is associated with improved outcome in rectal cancer patients who undergo chemoradiation therapy. Cancers.

[24] Giampieri R., Restivo A., Pusceddu V. (2017). The role of aspirin as antitumoral agent for heavily pretreated patients with metastatic colorectal cancer receiving capecitabine monotherapy. Clin Colorectal Cancer.

[25] Casadei-Gardini A., Rovesti G., Dadduzio V. (2021). Impact of aspirin on clinical outcome in advanced HCC patients receiving sorafenib and regorafenib. HPB (Oxford).

[26] Chun Y.S., Vauthey J.N., Boonsirikamchai P. (2009). Association of computed tomography morphologic criteria with pathologic response and survival in patients treated with bevacizumab for colorectal liver metastases. JAMA.

[27] Overs A., Flammang M., Hervouet E. (2021). The detection of specific hypermethylated WIF1 and NPY genes in circulating DNA by crystal digital PCR^TM^ is a powerful new tool for colorectal cancer diagnosis and screening. BMC Cancer.

[28] Jary M., Hasanova R., Vienot A. (2020). Molecular description of ANGPT2 associated colorectal carcinoma. Int J Cancer.

[29] Vienot A., Vernerey D., Bouard A. (2025). Stanniocalcin 1 in patients with refractory colorectal cancer treated with regorafenib: a post-hoc biomarker analysis of the TEXCAN and CORRECT trials. Cancer Res Commun.

[30] Adenis A., de la Fouchardiere C., Paule B. (2016). Survival, safety, and prognostic factors for outcome with Regorafenib in patients with metastatic colorectal cancer refractory to standard therapies: results from a multicenter study (REBECCA) nested within a compassionate use program. BMC Cancer.

[31] Lee D.-W., Lim Y., Kim H.P. (2023). Circulating tumor DNA dynamics and treatment outcome of regorafenib in metastatic colorectal cancer. Cancer Res Treat.

[32] Khan K., Rata M., Cunningham D. (2018). Functional imaging and circulating biomarkers of response to regorafenib in treatment-refractory metastatic colorectal cancer patients in a prospective phase II study. Gut.

[33] Tabernero J., Lenz H.J., Siena S. (2015). Analysis of circulating DNA and protein biomarkers to predict the clinical activity of regorafenib and assess prognosis in patients with metastatic colorectal cancer: a retrospective, exploratory analysis of the CORRECT trial. Lancet Oncol.

