# Supplementary figures and images for: Regorafenib and metronomic capecitabine, cyclophosphamide, and aspirin in refractory metastatic colorectal cancer: results from the REPROGRAM-01 single-arm phase II trial
Source: ESMO Gastrointest Oncol. 2025 Dec 3;10:100270. doi: 10.1016/j.esmogo.2025.100270 (PMC13332125; doi:10.1016/j.esmogo.2025.100270)

## Slide 1
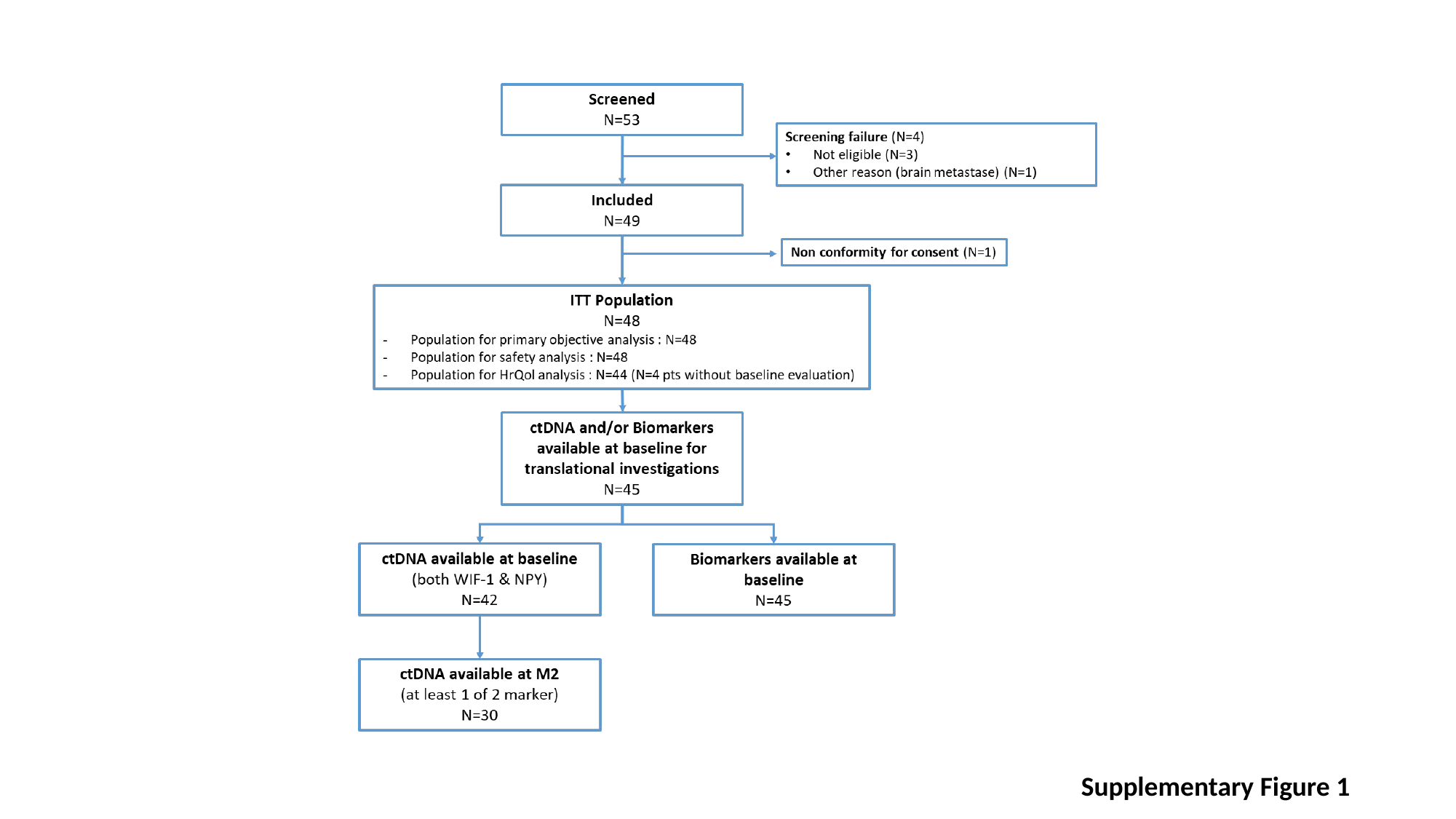

Supplementary Figure 1

Supplement: Supplementary Figure 1 [file mmc1.pptx]

## Slide 1
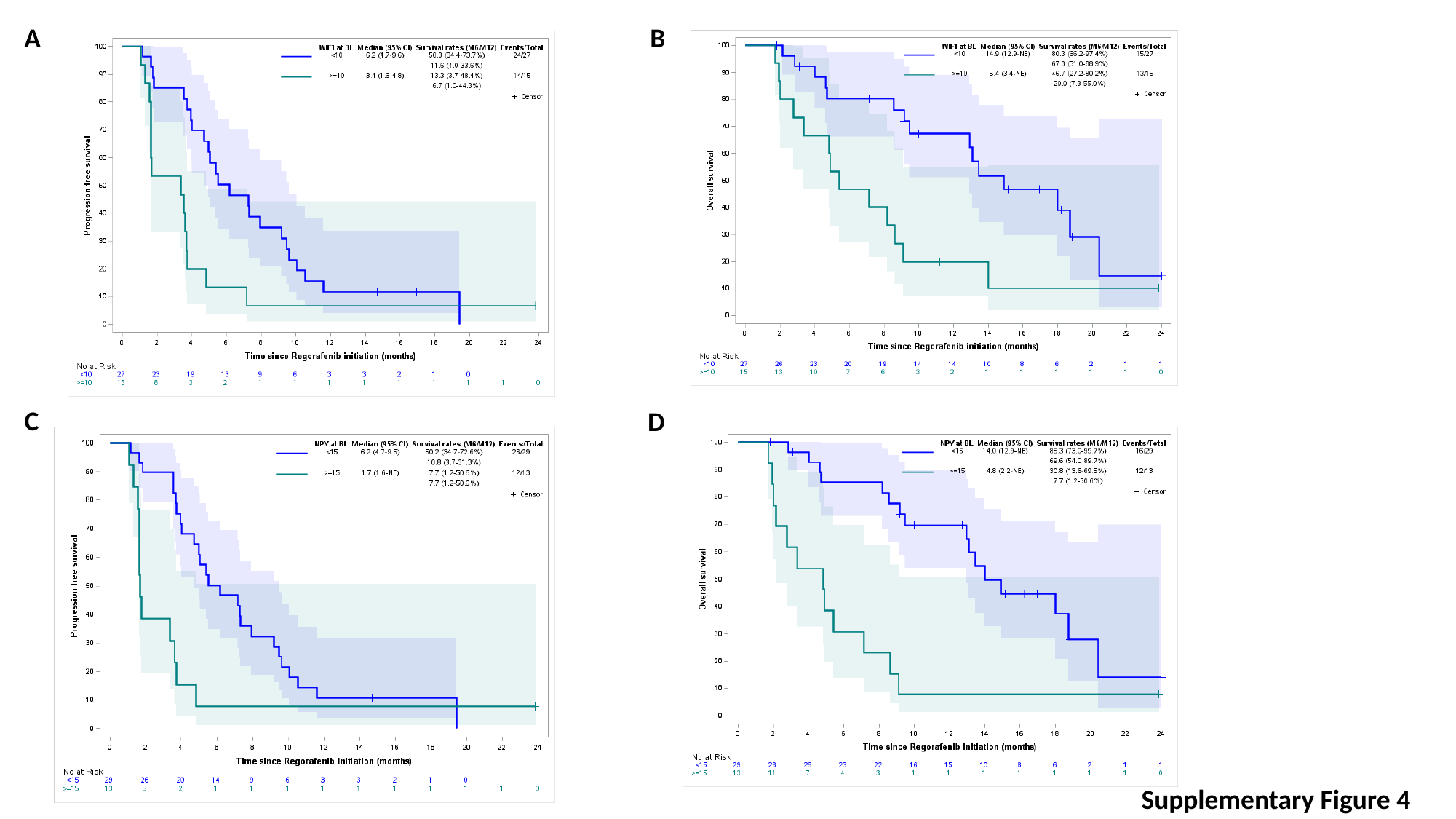

A
B
C
D
Supplementary Figure 4

Supplement: Supplementary Figure 4 [file mmc4.pptx]
