# Supplementary material for: Regorafenib and metronomic capecitabine, cyclophosphamide, and aspirin in refractory metastatic colorectal cancer: results from the REPROGRAM-01 single-arm phase II trial
Source: ESMO Gastrointest Oncol. 2025 Dec 3;10:100270. doi: 10.1016/j.esmogo.2025.100270 (PMC13332125; doi:10.1016/j.esmogo.2025.100270)
Supplement: Supplementary Figure 2 [file mmc2.docx]

**Supplementary Figure 2.**

1. B.


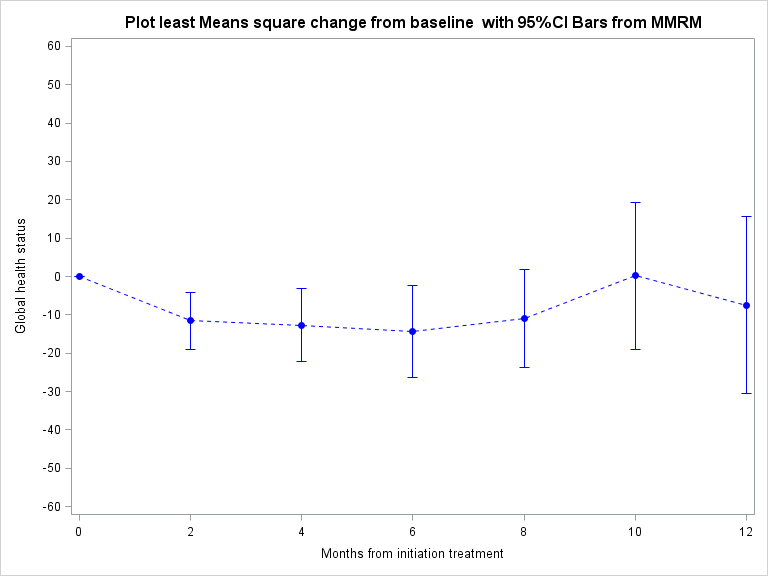

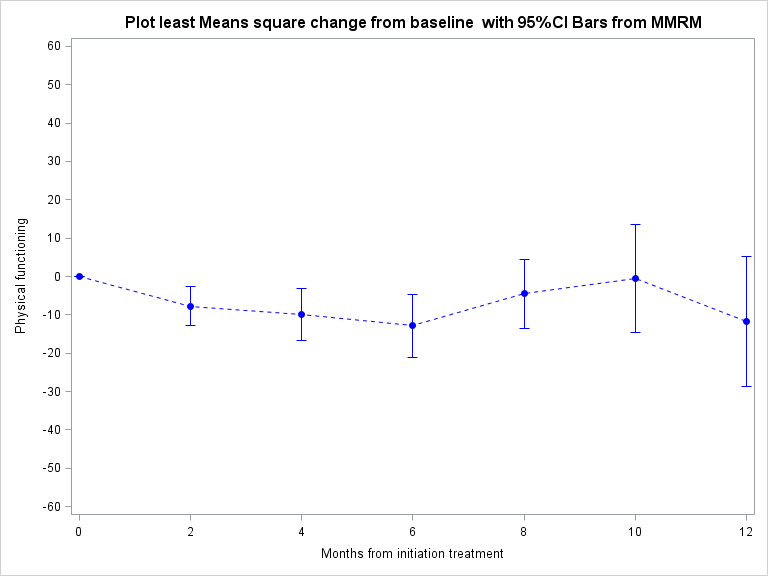


C. D.


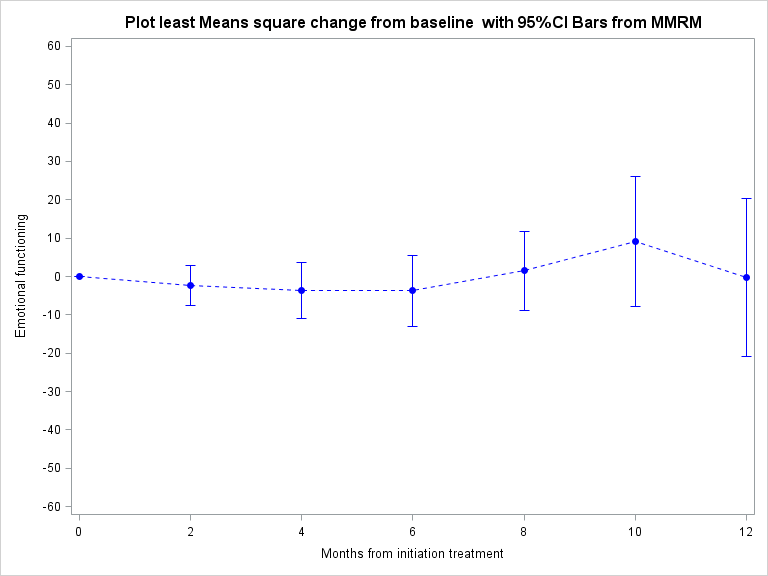

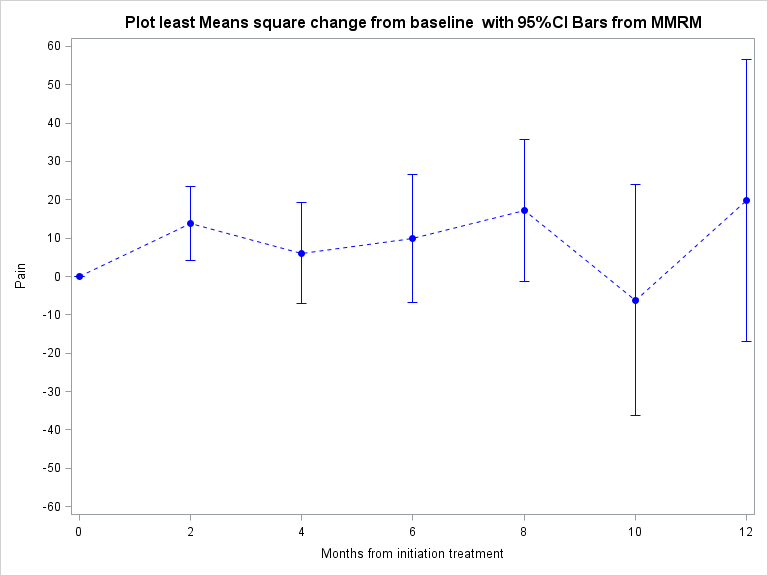


E.


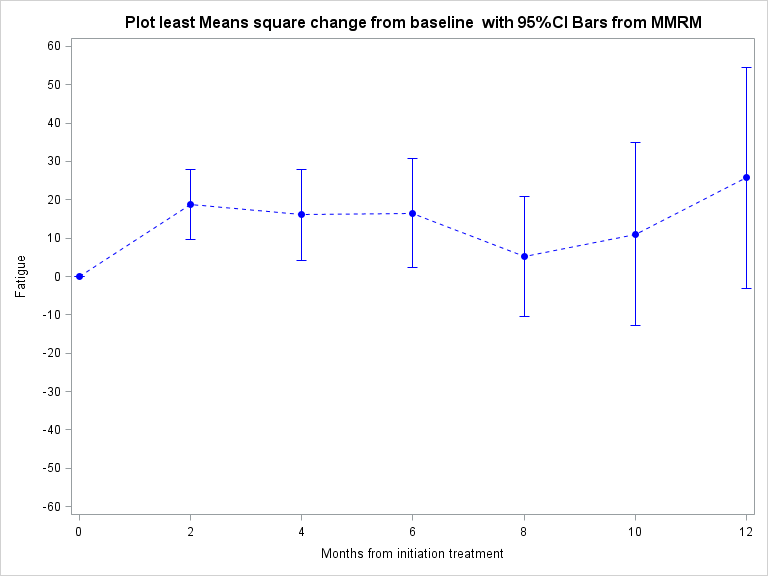


Footnote : A diminution of scores for global heath status , physical functioning , emotional functioning and an increase of score for pain and fatigue corresponds to a worse quality of life.

Abbreviation : MMRM ; Mixed model for repeated mesures , CI, confidence interval
