# Supplementary material for: Regorafenib and metronomic capecitabine, cyclophosphamide, and aspirin in refractory metastatic colorectal cancer: results from the REPROGRAM-01 single-arm phase II trial
Source: ESMO Gastrointest Oncol. 2025 Dec 3;10:100270. doi: 10.1016/j.esmogo.2025.100270 (PMC13332125; doi:10.1016/j.esmogo.2025.100270)
Supplement: Supplementary Figure 3 [file mmc3.docx]

**Supplementary Figure 3.**

1. B.


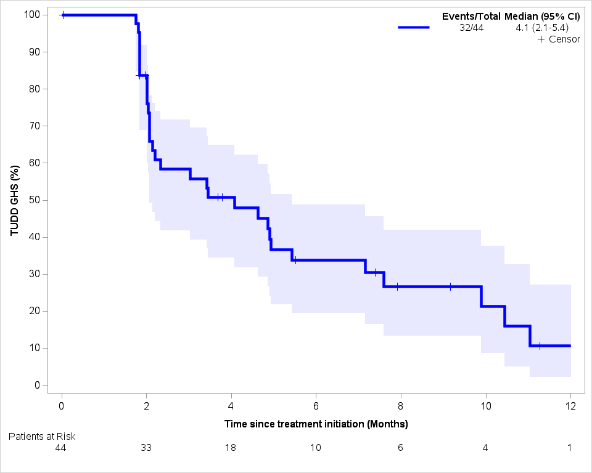

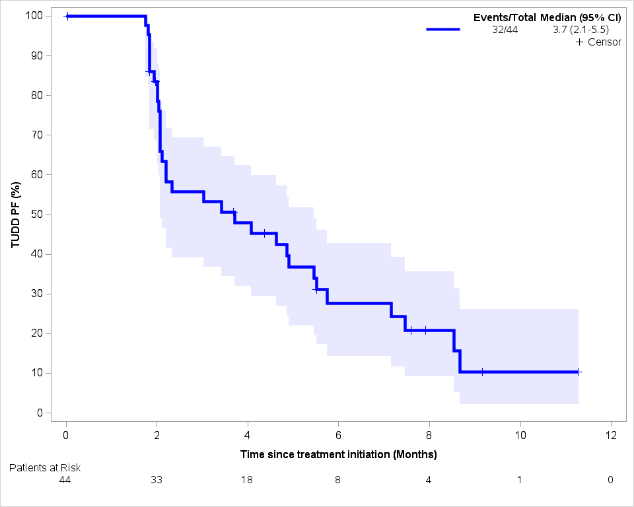


C. D.


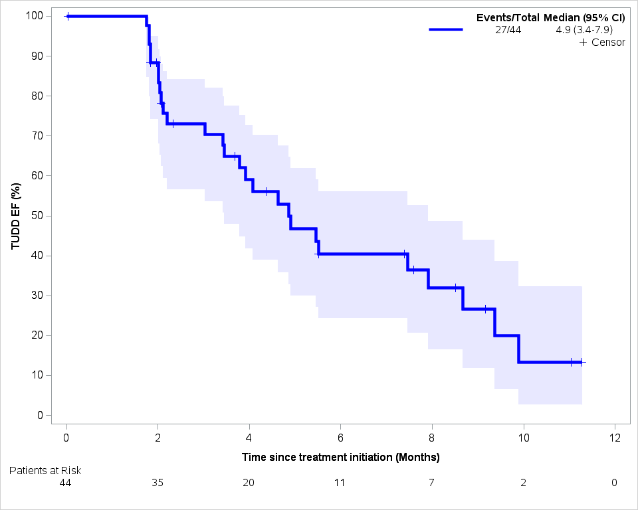


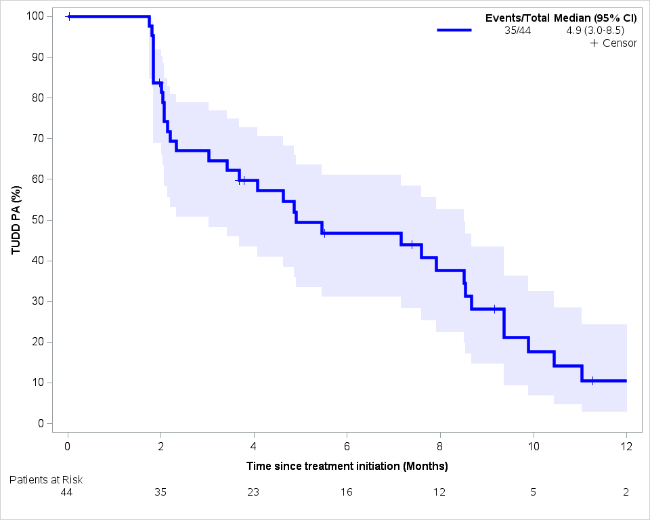


E.


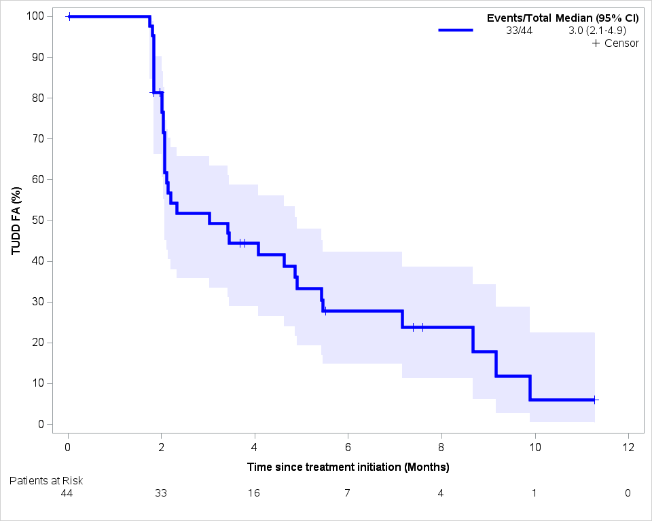


Abbreviation : Time until definitive deterioration, TUDD ; CI confidence interval ; MCID : minimal clinically important difference
