# Supplementary material for: Regorafenib and metronomic capecitabine, cyclophosphamide, and aspirin in refractory metastatic colorectal cancer: results from the REPROGRAM-01 single-arm phase II trial
Source: ESMO Gastrointest Oncol. 2025 Dec 3;10:100270. doi: 10.1016/j.esmogo.2025.100270 (PMC13332125; doi:10.1016/j.esmogo.2025.100270)
Supplement: Supplementary Data [file mmc5.docx]

**Supplementary figure legends**

**Supplementary Figure 1 Reprogram 01 study flow chart.**

Patient Flow with an illustration of the intention-to-treat, safety, and Response evaluation according to samples available for ctDNA and angiogenic-related biomarker analyses.

**Supplementary Figure 2. Cox proportional hazards for time to improvement/(end of) stable state/worsening and linear mixed models for repeated measures (MMRM) for the targeted quality of life dimensions observed in patients treated with regorafenib and multimodal metronomic chemotherapy.**

Plot of the least mean square change from baseline with 95% CI bars from MMRM for the targeted dimensions: global heath status (A), physical functioning (B), emotional functioning (C), pain (D), fatigue (E)

**Supplementary Figure 3. Time until definitive deterioration of the quality of life for patients treated with regorafenib and multimodal metronomic chemotherapy.**

Time until definitive deterioration (MCID of 5 points) or death for the targeted dimensions : global heath status (A) , physical functioning (B), emotional functioning (C) pain (D), fatigue (E)

**Supplementary Figure 4. Baseline WIF1 and NPY methylated ctDNA levels are correlated to survival of patients included in Reprogram01 study.**

The influence of baseline (BL) levels of WIF1 or NPY methylated ctDNA were investigated according to thresholds defined using restricted cubic spline method. Kaplan meier curves depicting the progression free and overall survival for patients according to BL WIF1 methylated ctDNA are shown in panel A and B. Kaplan meier curves depicting the progression free and overall survival for patients according to BL NPY methylated ctDNA are shown in panel C and D.
